# *BRCA1* and *BRCA2* mutations and clinical interpretation in 398 ovarian cancer patients: comparison with breast cancer variants in a similar population

**DOI:** 10.1186/s40246-018-0171-5

**Published:** 2018-08-13

**Authors:** Florencia C. Cardoso, Susana Goncalves, Pablo G. Mele, Natalia C. Liria, Leonardo Sganga, Ignacio Diaz Perez, Ernesto J. Podesta, Angela R. Solano

**Affiliations:** 1 Genotipificación y Cáncer Hereditario, Centro de Educación Médica e Investigaciones Clínicas “Norberto Quirno” (CEMIC), Galván 4102, C1431FWO Ciudad Autonoma de Buenos Aires, Argentina; 2 Instituto de Investigaciones Biomédicas (INBIOMED), Facultad de Medicina, Universidad de Buenos Aires-CONICET, Paraguay 2155 - Piso 5, C1121ABG Ciudad Autonoma de Buenos Aires, Argentina; 3 AstraZeneca Argentina MC, Vedia 3616, C1430DAH Ciudad Autonoma de Buenos Aires, Argentina

**Keywords:** Ovarian cancer, *BRCA1/2* and ovarian cancer, iPARP treatment

## Abstract

**Background:**

Ovarian cancer is the leading cause of death worldwide among gynecologic malignancies. The recent approval of inhibitors of poly (ADP-ribose) polymerase (iPARP) in the treatment of ovarian cancer in the presence of a *BRCA1/2* mutation has sparked the analysis of women with such diagnosis, which can further benefit from the detection of carriers in the family. Germline sequence and large rearrangements for *BRCA1/2* were tested in 398 consecutive epithelial ovarian cancer (EOC) patients.

The aim of this study was to identify the frequency and spectrum of germline *BRCA1/2* pathogenic alterations in a cohort of patients with ovarian serous carcinoma, with a view to adequately selecting patients for prevention through family counseling and correlating this frequency with platinum sensitivity as a guidance to identify patients eligible for iPARP in our population.

**Results:**

A total of 96 patients carried a pathogenic germline mutation, accounting for an overall 24.1% mutation incidence. Among mutation carriers, *BRCA1 showed* 62.5% incidence, *BRCA2* rendered 36.5%, and one patient exhibited a mutation in both genes. Three pathogenic mutations were recurrent mutations detected five, three, and four times and represented 12.5% of the mutated samples. Worth highlighting, a 50% mutation incidence was detected when breast and ovarian cancer coexisted in the same patient. Novel mutations amounted to 9.4% of the total mutations, as compared to 4.7% in breast cancer. Forty out of 60 *BRCA1* mutations were beyond the ovarian cancer cluster region (OCCR), in stark contrast with 22 out of 36 *BRCA2* mutations being inside the OCCR. Taken together, germline *BRCA1/2* mutations in EOC patients showed a distinct mutational spectrum compared to our previously published data on breast cancer patients.

**Conclusions:**

In sum, our study provides novel data on ovarian *BRCA1/2* mutation prevalence worldwide, enhances adequate patient selection for family counseling and prevention, and sheds light on the benefits of iPARP treatment.

**Electronic supplementary material:**

The online version of this article (10.1186/s40246-018-0171-5) contains supplementary material, which is available to authorized users.

## Background

Ovarian cancer is the leading cause of death worldwide among gynecologic malignancies. Argentina exhibits mid-high rates, and, in 2016, the National Cancer Institute, Ministry of Health (Instituto Nacional del Cáncer, INC, Ministerio de Salud de la Nación), reported a total of 2274 ovarian cancer cases out of a total of 60,209 women cancer cases, which represents 3.8% (Argentina, 2016, SIVER-Ca, INC, Ministerio de Salud de la Nación).

Genetic testing for *BRCA1/2* mutation carriers proves critical to clinical decisions, as more than 90% of the cases of epithelial ovarian cancer (EOC) are diagnosed with bulky intra-abdominal disease or distant metastases [1]. The importance of *BRCA1/2* mutation screening in ovarian cancer patients has been further underscored by recent findings showing that mutation carriers have increased sensitivity to inhibitors of poly (ADP-ribose) polymerase (PARP) [2, 3]. In fact, PARP inhibitors (iPARP) have recently been approved for the treatment of advanced ovarian cancer patients carrying either germline or somatic mutations in *BRCA1*/*2* genes [4]. Moreover, *BRCA1/2* mutation status has been shown to predict response to iPARP. Individuals with germline *BRCA1/2* alterations treated with iPARP have a significant increase in progression-free survival compared with patients with wild-type *BRCA1/2* [3, 5].

The reported prevalence of *BRCA1/2* mutations in patients with ovarian cancer varies across different studies and ethnic populations. A report interpreting the results of 14 studies from eight Western countries, summarized in a meta-analysis, has shown the overall incidence of germline mutations to be 18.0% for *BRCA1* and 6.9% for *BRCA2*, although this incidence ranges between 3.4 and 47% for *BRCA1* and between 1 and 12% for *BRCA2* [6] when considering specific populations. Reports from Asia have revealed the following figures: in South Korea [7], only one pathogenic mutation was found in the *BRCA1* gene among 37 EOC patients; a Japanese study [8] found 5.3% cases with germline mutations in *BRCA1* and 7.4% in *BRCA2*; in Hong Kong [9], the publication of a series of 60 ovarian cancer patients analyzed for the whole coding region of *BRCA1* but only the exon 11 of *BRCA2* rendered 11.3% and 2.1% patients carrying a mutation, respectively, including the c.1081delG in *BRCA1*, which seemed to be a founder mutation from Southern Chinese populations, and two recurrent mutations, i.e., c.2371-2372delTG in *BRCA1* and c.3337C>T in *BRCA2*; finally, in the Chinese population [10], the rate of mutation carriers among patients was reported to be 16.7%, with the description of a presumably very frequent non-founder mutation, i.e. c.5470_5477del8 in *BRCA1*, and the conclusion that the spectrum of *BRCA1/2* mutations greatly differs from that described in Western studies.

Relatively few studies have been reported on South American populations, and the most readily available results are based on small-size cohorts. A Colombian study has reported 100 patients with ovarian cancer diagnosis and 15% of mutation detection—13% in *BRCA1* and 2% in *BRCA2*—including an 11% accounting for a founder mutation [11]. A review of Latin American and Caribbean studies summarizes breast/ovarian cancer cases from a few countries. However, only a limited number of studies used full sequencing analysis and ovarian cancer was not clearly disclosed, which prevented the review from reaching conclusions [12].

To gain a more complete insight into the prevalence of *BRCA1/2* mutations in EOC patients from Argentina, we performed a cohort study of 398 unselected consecutive EOC patients for *BRCA1/2* mutation screening using the next-generation sequencing (NGS) approach and multiplex ligation-dependent probe amplification (MLPA) for large rearrangements.

The aim of this study was to identify the frequency and spectrum of germline *BRCA1/2* pathogenic alterations in a cohort of patients with ovarian serous carcinoma, with a view to adequately selecting patients for prevention through family counseling and correlating this frequency with platinum sensitivity as a guidance to identify patients eligible for iPARP in our population. In addition, and considering similarities in the population analyzed, this study presents a comparison with results previously published by our group [13] on *BRCA1/2* mutations in breast cancer patients.

## Methods

### Study subjects

Subjects were selected among women diagnosed with epithelial ovarian cancer and referred to Centro de Educación Médica e Investigaciones Clínicas (CEMIC) for genetic testing from January 2014 to June 2017. A total of 398 patients were included in the study, 299 of whom were selected by the inclusion criteria required for treatment with iPARP (high-grade ovarian serous carcinoma, relapsed, second-line platinum-sensitive). Routine procedure included signing a written informed consent to genetic testing (including anonymized disclosure of the data) from each patient, approved by the Ethics Committee from CEMIC, and a Pretest Counseling for Susceptibility Testing (purpose of testing), as described in the American Society of Clinical Oncology Policy Statement Update [14].

Subjects enrolled in this study showed a mean age at diagnosis of 53.5 ± 12 years, within a range of 18 to 84 years of age. Eligible patients included women with newly diagnosed, histologically confirmed, or chemotherapy-treated serous ovarian cancer, regardless of chemotherapy line. Although data on family history (FH) were collected as part of the study, recruitment was independent of FH conditions.

### *BRCA* testing

Genomic DNA of the 398 blood samples was isolated by MagNA Pure® LC instrument with total DNA isolation kit I (Roche Diagnostics). Analysis of *BRCA1/2* genes included complete sequencing and study of large rearrangements.

The Ion AmpliSeq*BRCA1/2* community panel was used for the targeted NGS, as it allows to amplify the entire coding sequences of *BRCA1* and *BRCA2*, including 20–50 bases of adjacent intronic sequence of each exon. Sequencing of the amplified regions was performed with the next-generation platform Personal Genome Machine® System. As a control, the STR variants of every sample were previously traced and intra NGS [15] was used to ensure the identification of the sample and avoid possible processing. The few codifying sequences with low readings were analyzed by Sanger reaction in order to reach 100% coverage.

The raw signal data and the sequence reads were processed with Ion Torrent Suite software (Thermo Fisher Scientific) on a Torrent server. The pipeline included signaling processing, base calling, quality score assignment, adapter trimming, PCR duplicate removal, read alignment to the reference human genome 19, quality control of mapping quality, coverage analysis, and variant calling. Coverage analysis used plug-in software in the Torrent server. The variant caller parameter setting was germline PGM (Life Technologies).

After data analysis, single-nucleotide variants, insertions, deletions, and splice site alternations were registered, and all variants detected were reported. Sanger DNA sequencing was used to confirm all clinically relevant variants detected (classes 3, 4, and 5) using the specific gene primers. Clinical significance was determined according to the report in the reference databases (ClinVar [16], LOVD 3.0 [17], UMD [18]—last access December 29, 2017). For missense mutations not reported or reported with uncertain clinical significance (VUS), in silico programs were used to predict the change in protein function using software Align-GVGD, SIFT, and Mutations Taster.

Large rearrangements were measured by MLPA using SALSA MLPA Probemix P002-D1 and P045-B3 provided by MRC-Holland, and Coffalyser.net software was used for data analysis.

In a preliminary analysis for a panel of genes, 30 samples were exome sequenced and then filtered for the following: *ATM*, *BRCA1*, *BRCA2*, *BRIP1*, *CDH1*, *CHEK2*, *MSH2*, *MLH1*, *MSH6*, *PMS2*, *EPCAM*, *NBN*, *NF1*, *PALB2*, *PTEN*, *RAD51C*, *RAD51D*, *STK11*, and *TP53*. Full exonic ± 20 bases of adjacent intronic sequence for each gene were assured. These genes were selected according to the genes listed in the NCCN guidelines (Genetic/Familial High-Risk Assessment: Breast and Ovarian—Version 2.2017), for which there are risk and management recommendations of patients with a pathogenic mutation. In other words, these actionable genes allow clinical measures such as monitoring, treatment, counseling, and prevention for both the probands and their families.

We routinely share our genetic variants and collected at Leiden Open Variation Database (Chapter for Argentina) [19]. In the case of the novel variants, the registration numbers of each of the variants in the LOVD database [17] are shown in Table 2.

### Genetic variant classification

The novel variants were classified according to the recommendation guidelines of the American College of Medical and Genomics (ACMG) [20]. As they correspond to variants not reported in the population and disease database all comply with the PM2 criteria of the ACMG Guidelines, this is a criterion of moderate pathogenicity. According to this:

Probably pathogenic mutations were defined as follows:

1. Nonsense and frameshift variants that generate a premature stop codon, except for the variants that generate a premature stop codon after codon 3326 in the BRCA2 gene (criterion PVS1 of the ACMG guidelines)
2. Splice site variants that are found in intronic or exonic variant in the exon-intron border (criterion PVS1 of the ACMG guidelines)

Variants of uncertain clinical significance (VUS) were defined as follows:

1. Missense variants where multiple lines of computational evidence support to deleterious effect on the gene or gene product or no impact on gene or gene product (criteria PP3 and BP4 respectively of ACMG guidelines)
2. Synonymous (silent) variant for which splicing prediction algorithms predict no impact to the splice consensus sequence nor the creation of a new splice site and the nucleotide is not highly conserved (criterion BP7 ACMG guidelines)
3. Intronic variants distant from the intron-exon boundary

## Results

The sequencing of *BRCA1/2* in 398 consecutive EOC patients, including 299 patients selected for iPARP treatment, rendered a total of 96 patients carrying a pathogenic germline mutation. These cases are listed in Additional file 1, which indicates patients selected for iPARP treatment in italics and patients with novel mutations in bold. Overall mutation incidence amounted to 24.1% (*n* = 398), while mutation incidence among patients selected for iPARP treatment was 20.7% (*n* = 299) and mutation incidence among non-iPARP-selected patients was 34.3% (*n* = 99).

The mutations found were as follows: 60 in *BRCA1* (62.5%), 35 in *BRCA2* (36.5%), and 1 in both genes (1%) (Table 1). The mean age of diagnosis for the patients carrying a mutation was 53.7 years, which was non-statistically different from 54.3 years in the non-detected-mutation group. The low end of the age range among patients with a non-detected mutation, as well as among patients with both breast and ovary cancer diagnosis was 31 years of age, much higher than that of the mutation-carrying group at 18 years (Table 1). Remarkably, the patients diagnosed with both cancers showed a 50% rate of mutation detection (Table 1), again with similar distribution in both genes: 69% detected in *BRCA1*, 27.6% in *BRCA2*, and 3.4% in both genes.

**Table 1:** *BRCA1/*2 sequence: summary of patients analyzed

Diagnosis	*n*	Age (range)^a^	BRCA mutated (%)	*BRCA1* mutation carriers (*n*)	*BRCA2* mutation carriers (*n*)	Non mutated (%)
EOC (total)	398	53.5 (18–84)	96 (24.1)	61^b^	36^b^	302 (75.9)
EOC and BC	58	56.5 (31–78)	29 (50.0)	21^b^	9^b^	29 (50.0)

The total number of patients analyzed was diagnosed with epithelial ovarian cancer (398). Among these, 58 patients also had a diagnosis of breast cancer (EOC and BC)

*EOC* epithelial ovarian cancer, *BC* breast cancer, *n* number of cases

^a^Age at ovary cancer diagnosis

^b^One of the patients with high-grade ovarian serous carcinoma and breast cancer had a mutation in both genes

Key to validating our population detection methods and interpretation, only 3.38% variants were found of unknown significance (VUS). Out of the total number of mutations detected, nine were found to be novel deleterious mutations (9.4%), three of them in *BRCA1* and six in *BRCA2*, as listed in Table 2. Of note, *BRCA1* mutation c.3578_3759delCT has been previously described by our group in an unrelated patient [21], although this patient had breast cancer diagnosed at 31 years; *BRCA2* mutation c.7805+2_7805+3delTA has also been already described [13] but is included now as belonging to an ovary cancer patient. Novel intronic variants detected to be yet classified were c.670+31A>C, c.4357+22C>T, c.80+52T>A, and c.516+3A>T.

**Table 2:** Novel variants in *BRCA1* (NM_007294.3) and *BRCA2* (NM_000059.3) genes detected in 398 probands with diagnosis of epithelial ovarian cancer

Sample ID	Gene	Exon/intron	Mutation^a^	Predicted effect^b^	MT	CS^c^	LOVD (genomic variant #)
BR1229	1B	11	c.2005dupA	p.(Met669Asnfs*4)	F	LP	198881
BR2066	1B	11	c.3758_3759delCT	p.(Ser1253*)	F	LP	196851
BR1037	1B	11	c.876_879delCACT	p.(Thr293Lysfs*4)	F	LP	198751
BR1410	2B	11	c.2133C>A	p.(Cys711*)	N	LP	202233
BR0986	2B	11	c.2860G>T	p.(Glu954*)	N	LP	197664
BR0832	2B	11	c.4419delC	p.(Asn1473Lysfs*6)	F	LP	201398
BR2072	2B	11	c.5253C>A	p.(Tyr1751*)	N	LP	203562
BR1464	2B	14	c.7308delC	p.(Asn2436Lysfs*31)	F	LP	206927
BR0495	2B	16i	c.7805+2_7805+3delTA		S	LP	199222
BR1104	1B	11	c.2357T>C	p.(Leu786Pro)	M	VUS	200898
BR0889	1B	11	c.3168C>T	p.(Ser1056=)	Syn	VUS	200773
BR1061	1B	10i	c.670+31A>C		S	VUS	196392
BR2063	1B	13i	c.4357+22C>T		S	VUS	209404
BR1078	1B	2i	c.80+52T>A		S	VUS	196406
BR0913	2B	6i	c.516+3A>T		S	VUS	199956

*MT* mutation type, *F* frameshift, *N* nonsense, *S* splicing, *M* missense, *Syn* synonym, *LP* likely pathogenic, *VUS* variant of uncertain significance

^a^HGVS nomenclature at cDNA level

^b^HGVS nomenclature at protein level

^c^CS: interpretation and classification of the variants was carried out according to the recommendations of the ACMG guidelines

Regarding recurrent pathogenic mutations, present in 3 or more patients each, 2 were detected in *BRCA1* and 1 in *BRCA2*, including 12 patients among mutation carriers (12.5%) (Table 3). These recurrent mutations were c.4964_4982del19 and c.5266dupC in *BRCA1* and c.5351dupA in *BRCA2*, with five, three, and four detections, respectively.

**Table 3:** Recurrent mutations in *BRCA1/2* detected in 398 probands with epithelial ovarian cancer

Mutation/times detected	Unrelated probands (% of the total probands)
*BRCA1*	
c.4964_4982del19 - p.(Ser1655Tyrfs*16)/5	5 (1.3)
c.5266dupC - p.(Gln1756Profs*74)/3	3 (0.8)
*BRCA2*	
c.5351dupA - p.(Asn1784Lysfs*3)/4	4 (1.0)
Total recurrent	12 (3.1)
Total recurrent (12)/total mutated (96) = 12.5%	–

The FH group—understood as having at least 1 relative developing breast and/or ovarian cancer among first- or second-degree relatives—accounted for 158 cases, among which 55 rendered mutations (34.8%), including the patient with a mutation in both genes. In turn, the non-FH group included 105 patients, 10 of whom were mutation carriers (9.5%). No FH records were available for the remaining 135 patients, 31 of whom revealed a mutation (23.0%) (Table 4).

**Table 4:** Family history in patients with epithelial ovarian cancer

Family history	Number of probands (% of total)	Patients with a mutation detected (%)
Yes^a^	158 (39.7)	55 (34.8)
No	105 (26.4)	10 (9.5)
Not known	135 (33.9)	31 (23.0)

^a^Yes: family history with at least 1 relative developing breast and/or ovarian cancer among first- or second-degree relatives

**Fig. 1:**
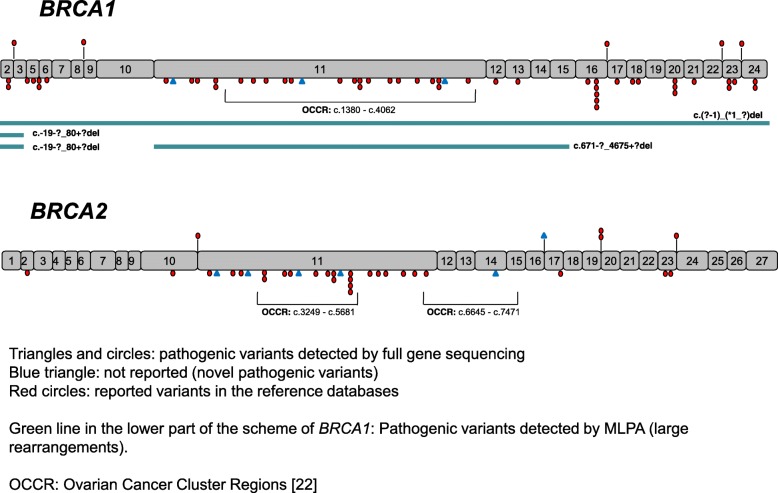
Schematic representation of the 96 BRCA1/2 pathogenic mutations detected in 398 patients with diagnosis of epithelial ovarian cancer

Regarding the spectrum of mutations along the genes (Fig. 1), 40 out of 60 mutations detected in *BRCA1* were located outside the ovarian cancer cluster region (OCCR), in contrast with the findings for *BRCA2*, in which 22 out of 36 were located inside the OCCR. In the case of the patient carrying a mutation in the two genes, both mutations were outside the OCCR. These mutations were c.-19-?_80+?del and c.1909+1G>A for *BRCA1* and *BRCA2*, respectively (Additional file 1).

## Discussion

In the current study, we have assessed the *BRCA1/2* mutation status in 398 EOC patients with two main goals: the benefits of detecting hereditary breast/ovarian cancer syndrome for prevention and the possibility of selecting patients for treatment with iPARP. Results showed most mutations to be found in the *BRCA1* gene, reinforcing once again the well-established association of ovary cancer and a mutation in *BRCA1*. In our series, however, most of the mutations were outside the region of OCCR of *BRCA1*, even as the most recent and detailed publication [22] reinforcing the necessity of reporting regional genetic variants [19] and depositing genetic variants in open access databases. The apparent differences with the reported data [22] may very likely due to that no data was included from South America (or may be very little hidden in one of the categories); it is important to remark that the Hispanic demographic group in this very wide population analysis refers to a migration denomination in the USA and does not reflect South America and specifically, our country [13].

Our assay is a comprehensive analysis, and our group has vast experience in the regionality of the mutation spectra in our patients [13, 23], which is reflected in the low rate of 3.38% of VUS found in this series. The frequency of mutations detected reached 24.1% (Table 1), a value closer to the highest described in the literature [24, 25], lower than other published results [8, 11, 26–31]. The 99 patients not selected for iPARP treatment showed a striking proportion of 34.3% mutation carriers, which may stem from the fact that CEMIC is a reference center for hereditary breast-ovary cancer patient analysis.

The worldwide age range of patients diagnosed with EOC included in *BRCA1/2* analyses [8, 11, 24–32] starts around 30 years, with the exception of Colombia [11] (16 years) and Argentina [13] (18 years). The lower-end value of the range does not reflect the most frequent age of diagnosis, as cases diagnosed in the patient’s sixties are common, frequently with a mutation detected. This is reflected in the similar mean age among all the results published.

The high 50% rate of *BRCA1/2* mutations detected in 58 patients with both cancers diagnosis (Table 1) is in line with other studies on similar patients, although in smaller numbers, such as the Japanese study with 3 cases [8], all with a mutation detected. This is also in agreement with our previous publication [13], in which we analyzed 14 patients with breast and ovary cancer, 11 of whom (78.6%) bore a pathogenic mutation. Interestingly, seven mutations were found in common with the current series of patients, as follows: c.211A>G, c.1687C>T, c.1892dupT, c.5266dupC, c.5468-1G>A, c.2808_2811delACAA, and c.5351dupA (Additional file 1).

Worth pointing out, the gene spectrum (Fig. 1), including data on the OCCR and the recurrent mutations described (Table 3), does not visualize a panel or hot spot of mutations to abbreviate the analysis of *BRCA1/2* in our ovarian cancer population.

As an additional comment regarding FH (not included in the criteria for the selection of patients), complementary data in the analysis of the results reveals large differences obtained in mutation detection between the FH and non-FH groups (34.8% vs 9.5%, *p* value = 0.000003, significant at *p* < 0.05), which is in contrast with results previously published [8]. This discrepancy may be explained by the larger number of patients studied in our report (398 vs 95), and our higher rate of mutations detected (24.1% vs 12.6%).

In turn, the following observations when comparing the findings described for ovarian cancer patients with our previous publication including a vast majority of breast cancer patients [13] are as follows: (a) the rate of mutation detection was higher in ovarian cancer patients with 24.1% vs breast cancer with 19.04% (*p* value = 0.035611, significant at *p* < 0.05); (b) the rate of novel mutations showed a tendency: 9.4% for ovarian cancer vs 4.7% for breast cancer. The population analyzed was 398 subjects for ovary cancer and 940 subjects for breast cancer with a detection rate of 2.26% (9 out 398) and 0.85% (8 out 940) novel variants, respectively (*p* value = 0.035249, significant at *p* < 0.05); (c) the rate of recurrent mutations was similar for both groups. Interestingly, the spectrum of recurrent mutations for both genes was spread along both genes.

A promising turn in the treatment of ovarian cancer has been the attempt at repairing double-strand DNA damage by homologous recombination repair pathway (HRR) mechanisms. *BRCA1* and *BRCA2* are genes centrally involved in this process, and mutations resulting in damaged *BRCA1* or *BRCA2* proteins can lead to various types of cancer such as breast, ovarian, or prostate cancer among the most closely associated. Even in the presence of a pathogenic mutation in *BRCA*, single-strand break repair by non-homologous end joining is an alternative pathway to repair double-strand breaks, avoiding cell death pathways like apoptosis. iPARP cause HRR leading gene-deficient (including *BRCA1/2*) cancer cells to die by apoptosis. This is known as “synthetic lethality,” a concept developed upon evidence on sensitivity of *BRCA1/2* defective cells to platinum salts [33, 34].

Preliminary results from exome analysis of a panel of genes (see the “Methods” section) in 30 patients showed the following non-*BRCA1/2* mutations: in *EPCAM*, c.412C>T p.(Arg138*), and coexisting mutations as follows: in *MUTYH*, c.1105delC - p.(Ala371Profs*23) and in heterozygosis and *RAD51D*, c.1A>G p.(Met1Val). The application of these results is still under consideration, although the involvement of the *RAD51D* gene in the HRR mechanism should be highlighted.

## Conclusion

In sum, the strength of our study lies in the inclusion of 299 patients exclusively selected for treatment with iPARP, plus an extra of 99 cases which could also benefit from treatment, the use of thorough methodology and knowledge of our population regional variants [13, 23], as supported by the 3.38% of VUS found. The findings reported here thus offer *BRCA* mutation carriers the benefit of treatment possibilities and allow precise identification of hereditary breast-ovary disease and the preventive measures associated.

## Additional file

Additional file 1: Pathogenic mutations in *BRCA1* (NM_007294.3) and *BRCA2* (NM_000059.3) genes (*n* = 96) detected in 398 probands with diagnosis of epithelial ovarian cancer. (DOCX 31 kb)

## Abbreviations

EOC: Epithelial ovarian cancer
NGS: Next-generation sequencing
OCCR: Ovarian cancer cluster region
PARP inhibitors: Poly (ADP-ribose) polymerase inhibitors
