# Supplementary material for: BRCA1 and BRCA2 mutations and clinical interpretation in 398 ovarian cancer patients: comparison with breast cancer variants in a similar population
Source: Hum Genomics. 2018 Aug 13;12:39. doi: 10.1186/s40246-018-0171-5 (PMC6090818; doi:10.1186/s40246-018-0171-5)
Supplement: Supplementary file 1 — Pathogenic mutations in BRCA1 (NM_007294.3) and BRCA2 (NM_000059.3) genes (n = 96) detected in 398 probands with diagnosis of epithelial ovarian cancer. (DOCX 31 kb) [file 40246_2018_171_MOESM1_ESM.docx]

**Additional file 1**. Pathogenic mutations in *BRCA1* (NM_007294.3) and *BRCA2* (NM_000059.3) genes (n=96) detected in 398 probands with diagnosis of epithelial ovarian cancer.

| **Sample ID** | **BRCA** | **Exon/ Intron** | **Mutation^1^** | **Predicted effect^2^** | **MT^3^** | **BOC YES/NO^4^** |
| --- | --- | --- | --- | --- | --- | --- |
| BR1477 | BRCA1 | 2 | c.68_69delAG | p.(Glu23Valfs*17) | F | YES |
| BR0882 | BRCA1 | 2 | c.68_69delAG | p.(Glu23Valfs*17) | F | NO |
| BR1063 | BRCA1 | 5 | c.181T>G | p.(Cys61Gly) | M | NO |
| BR0991 | BRCA1 | 5 | c.190T>C | p.(Cys64Arg) | M | YES |
| BR0264 | BRCA1 | 5 | c.211A>G | p.(Arg71Gly) | M | NO |
| BR0715 | BRCA1 | 5 | c.211A>G | p.(Arg71Gly) | M | YES |
| BR0976 | BRCA1 | 6 | c.250G>T | p.(Glu84*) | N | NO |
| BR0578 | BRCA1 | 11 | c.1054G>T | p.(Glu352*) | N | YES |
| BR0604 | BRCA1 | 11 | c.1067delA | p.(Gln356Argfs*18) | F | YES |
| BR1151 | BRCA1 | 11 | c.1360_1361delAG | p.(Ser454*) | F | YES |
| BR1152 | BRCA1 | 11 | c.1360_1361delAG | p.(Ser454*) | F | NO |
| BR0957 | BRCA1 | 11 | c.1504_1507delTTAA | p.(Leu502Serfs*29) | F | NO |
| BR0240 | BRCA1 | 11 | c.1687C>T | p.(Gln563*) | N | YES |
| BR0960 | BRCA1 | 11 | c.1729_1730delGA | p.(Glu577Ilefs*8) | F | NO |
| BR0878 | BRCA1 | 11 | c.1817delC | p.(Pro606Leufs*6) | F | NO |
| BR0408 | BRCA1 | 11 | c.1892dupT | p.(Ser632Lysfs*4) | F | YES |
| **BR1229** | **BRCA1** | **11** | **c.2005dupA** | **p.(Met669Asnfs*4)** | **F** | **NO** |
| BR2203 | BRCA1 | 11 | c.2411_2412delAG | p.(Gln804Leufs*5) | F | NO |
| BR1094 | BRCA1 | 11 | c.2486_2487delTT | p.(Phe829*) | F | NO |
| BR1345 | BRCA1 | 11 | c.2487delT | p.(Phe829Leufs*17) | F | NO |
| BR0926 | BRCA1 | 11 | c.2487delT | p.(Phe829Leufs*17) | F | NO |
| BR1012 | BRCA1 | 11 | c.2568T>G | p.(Tyr856*) | N | NO |
| BR2040 | BRCA1 | 11 | c.2800C>T | p.(Gln934*) | N | YES |
| BR0552 | BRCA1 | 11 | c.2901_2902dupTC | p.(Pro968Leufs*33) | F | YES |
| BR2039 | BRCA1 | 11 | c.3309dupT | p.(Lys1104*) | F | NO |
| BR2054 | BRCA1 | 11 | c.3700_3704delGTAAA | p.(Val1234Glnfs*8) | F | YES |
| BR1000 | BRCA1 | 11 | c.3756_3759delGTCT | p.(Ser1253Argfs*10) | F | NO |
| BR2093 | BRCA1 | 11 | c.3756_3759delGTCT | p.(Ser1253Argfs*10) | F | NO |
| **BR2066** | **BRCA1** | **11** | **c.3758_3759delCT** | **p.(Ser1253*)** | **F** | **NO** |
| BR0838 | BRCA1 | 11 | c.4042G>T | p.(Gly1348*) | N | NO |
| BR0971 | BRCA1 | 11 | c.835delC | p.(His279Metfs*19) | F | NO |
| **BR1037** | **BRCA1** | **11** | **c.876_879delCACT** | **p.(Thr293Lysfs*4)** | **F** | **NO** |
| BR1330 | BRCA1 | 12 | c.4128_4129delAA | p.(Ser1377Argfs*3) | F | YES |
| BR1323 | BRCA1 | 13 | c.4327C>T | p.(Arg1443*) | N | YES |
| BR0974 | BRCA1 | 16 | c.4688dupA | p.(Tyr1563*) | F | NO |
| BR0610 | BRCA1 | 16 | c.4964_4982del19 | p.(Ser1655Tyrfs*16) | F | YES |
| BR0635 | BRCA1 | 16 | c.4964_4982del19 | p.(Ser1655Tyrfs*16) | F | NO |
| BR1349 | BRCA1 | 16 | c.4964_4982del19 | p.(Ser1655Tyrfs*16) | F | NO |
| BR0824 | BRCA1 | 16 | c.4964_4982del19 | p.(Ser1655Tyrfs*16) | F | YES |
| BR1503 | BRCA1 | 16 | c.4964_4982del19 | p.(Ser1655Tyrfs*16) | F | NO |
| BR0906 | BRCA1 | 17 | c.5030_5033delCTAA | p.(Thr1677Ilefs*2) | F | NO |
| BR1245 | BRCA1 | 18 | c.5095C>T | p.(Arg1699Trp) | M | NO |
| BR1092 | BRCA1 | 18 | c.5123C>A | p.(Ala1708Glu) | M | YES |
| BR2113 | BRCA1 | 20 | c.5266dupC | p.(Gln1756Profs*74) | F | NO |
| BR0613 | BRCA1 | 20 | c.5266dupC | p.(Gln1756Profs*74) | F | NO |
| BR0958 | BRCA1 | 20 | c.5266dupC | p.(Gln1756Profs*74) | F | YES |
| BR0608 | BRCA1 | 21 | c.5319dupC | p.(Asn1774Glnfs*56) | F | YES |
| BR0061 | BRCA1 | 23 | c.5434C>G | p.(Pro1812Ala) | M | NO |
| BR0927 | BRCA1 | 23 | c.5434C>G | p.(Pro1812Ala) | M | NO |
| BR1132 | BRCA1 | 23 | c.5444G>A | p.(Trp1815*) | N | NO |
| BR0082 | BRCA1 | 24 | c.5509T>C | p.(Trp1837Arg) | M | NO |
| BR1042 | BRCA1 | 24 | c.5509T>C | p.(Trp1837Arg) | M | NO |
| BR0250 | BRCA1 | 11-15 | c.671-?_4675+?del |  | LR | NO |
| BR1107 | BRCA1 | 1-24 | c.(?-1)_(*1_?)del |  | LR | NO |
| BR0859 | BRCA1 | 16i | c.4987-1G>A |  | S | NO |
| BR0699 | BRCA1 | 22i | c.5407-2A>G |  | S | NO |
| BR0002 | BRCA1 | 23i | c.5468-1G>A |  | S | YES |
| BR1011 | BRCA1 | 2i | c.81-1G>A |  | S | YES |
| BR0507 | BRCA1 | 5´UTR-2 | c.-19-?_80+?del |  | LR | NO |
| BR0937 | BRCA1 | 8i | c.547+2T>A |  | S | NO |
| BR1179 | BRCA1 | 5´UTR-2 | c.-19-?_80+?del |  | LR | YES |
|  | BRCA2 | 10i | c.1909+1G>A |  | S |  |
| BR1482 | BRCA2 | 2 | c.51_52delAC | p.(Arg18Leufs*12) | F | YES |
| BR1119 | BRCA2 | 10 | c.1597delA | p.(Thr533Leufs*25) | F | NO |
| BR1194 | BRCA2 | 11 | c.2094delA | p.(Gln699Serfs*31) | F | NO |
| **BR1410** | **BRCA2** | **11** | **c.2133C>A** | **p.(Cys711*)** | **N** | **NO** |
| BR2035 | BRCA2 | 11 | c.2657delA | p.(Asn886Metfs*9) | F | NO |
| BR2036 | BRCA2 | 11 | c.2808_2811delACAA | p.(Ala938Profs*21) | F | NO |
| **BR0986** | **BRCA2** | **11** | **c.2860G>T** | **p.(Glu954*)** | **N** | **NO** |
| BR0558 | BRCA2 | 11 | c.3545_3546delTT | p.(Phe1182*) | F | NO |
| BR2055 | BRCA2 | 11 | c.3545_3546delTT | p.(Phe1182*) | F | NO |
| BR0825 | BRCA2 | 11 | c.4222C>T | p.(Gln1408* ) | N | NO |
| BR2190 | BRCA2 | 11 | c.4284dupT | p.(Gln1429Serfs*9) | F | NO |
| **BR0832** | **BRCA2** | **11** | **c.4419delC** | **p.(Asn1473Lysfs*6)** | **F** | **NO** |
| BR2106 | BRCA2 | 11 | c.4964dupA | p.(Tyr1655*) | F | NO |
| BR1117 | BRCA2 | 11 | c.5130_5133delTGTA | p.(Tyr1710*) | F | NO |
| BR0951 | BRCA2 | 11 | c.5146_5149delTATG | p.(Tyr1716Lysfs*8) | F | NO |
| BR1111 | BRCA2 | 11 | c.5146_5149delTATG | p.(Tyr1716Lysfs*8) | F | NO |
| **BR2072** | **BRCA2** | **11** | **c.5253C>A** | **p.(Tyr1751*)** | **N** | **YES** |
| BR0029 | BRCA2 | 11 | c.5351dupA | p.(Asn1784Lysfs*3) | F | YES |
| BR1081 | BRCA2 | 11 | c.5351dupA | p.(Asn1784Lysfs*3) | F | NO |
| BR1120 | BRCA2 | 11 | c.5351dupA | p.(Asn1784Lysfs*3) | F | NO |
| BR1129 | BRCA2 | 11 | c.5351dupA | p.(Asn1784Lysfs*3) | F | NO |
| BR2047 | BRCA2 | 11 | c.5946delT | p.(Ser1982Argfs*22) | F | NO |
| BR1013 | BRCA2 | 11 | c.5959C>T | p.(Gln1987*) | N | NO |
| BR1153 | BRCA2 | 11 | c.6024dupG | p.(Gln2009Alafs*9) | F | YES |
| BR1350 | BRCA2 | 11 | c.6395T>G | p.(Leu2132*) | N | NO |
| BR1093 | BRCA2 | 11 | c.6596delC | p.(Thr2199Ilefs*7) | F | NO |
| BR0901 | BRCA2 | 11 | c.6833_6837delTCTTA | p.(Ile2278Serfs*13) | F | YES |
| **BR1464** | **BRCA2** | **14** | **c.7308delC** | **p.(Asn2436Lysfs*31)** | **F** | **YES** |
| BR2155 | BRCA2 | 17 | c.7976G>A | p.(Arg2659Lys) | M | NO |
| BR1230 | BRCA2 | 23 | c.8987T>A | p.(Leu2996*) | N | NO |
| BR1100 | BRCA2 | 23 | c.9026_9030delATCAT | p.(Tyr3009Serfs*7) | F | YES |
| BR0996 | BRCA2 | 19i | c.8488-1G>T |  | S | NO |
| BR0876 | BRCA2 | 19i | c.8488-1G>T |  | S | NO |
| **BR0495** | **BRCA2** | **16i** | **c.7805+2_7805+3delTA** |  | **S** | **NO** |
| BR1359 | BRCA2 | 23i | c.9118-1G>A |  | S | YES |

In bold: Novel pathogenic mutations (n=9), also listed in Table 4 of the report.

Sample BR1179 has 2 pathogenic mutations, one in BRCA1 and one in BRCA2.

Published mutations^15^: c.211A>C; c.1687C>T; c.1892dupT; c.5266dupC; c.5468-1G>A; c.2808_2811delACAA; c.5351dupA and c.5946delT

^1^ HGVS nomenclature at cDNA level

^2^ HGVS nomenclature at protein level

^3^Mutation type: F: Frameshift mutation; **N**: Nonsense mutation; **S**: Splicing mutation; **M**: Missense mutation; **LR**: Large Rearrangement mutation

^4^ Proband with diagnosis of breast and ovarian cancers
